# Supplementary material for: A Curated Rotamer Library for Common Post-Translational Modifications of Proteins
Source: ArXiv. 2024 May 6:arXiv:2405.03120v1. Preprint. [Version 1] (PMC11100909)
Supplement: 1 [file NIHPP2405.03120V1-supplement-1.pdf]

Supplementary Information
A Curated Rotamer Library for Common Post-Translational Modifications of Proteins

Oufan Zhang[1], Shubhankar A. Naik[2], Zi Hao Liu[5,6], Julie Forman-Kay[5,6], Teresa Head-Gordon[1-4*]

[1]Kenneth S. Pitzer Center for Theoretical Chemistry, [2]Department of Chemistry, [3]Department of Bioengineering, and [4]Department of Chemical and Biomolecular Engineering, University of California, Berkeley, Berkeley, California 94720, USA

[5]Molecular Medicine Program, Hospital for Sick Children, Toronto, Ontario M5G 0A4, Canada, [6]Department of Biochemistry, University of Toronto, Toronto, Ontario M5S 1A8, Canada

Supplementary Tables

Table S1. *Number of PDB files for PTM-modified proteins and their resolution ranges.* PDB files were found for phosphorylated serine (SEP), phosphorylated threonine (TPO), phosphorylated tyrosine (PTR), methylated arginine (AGM), mono-methylated lysine (MLZ), di-methylated lysine (MLY), tri-methylated lysine (M3L), oxidized methionine (OMT), and acetylated lysine (ALY). All PDB identifiers are provided in Table S2. Based on a trade-off between data quality and data abundance, we have only developed rotamer libraries for SEP, TPO, PTR, M3L, and ALY. See Methods for more details.

**Refinement Resolution (Å)**	**SEP**	**TPO**	**PTR**	**AGM**	**MLZ**	**MLY**	**M3L**	**OMT**	**ALY**
**1.0 - 1.5**	6	2	4	6	2	1	2	——	1
**1.5 - 2.0**	68	57	37	4	11	5	33	——	19
**2.0 - 2.5**	115	132	84	——	3	5	20	——	10
**2.5 - 3.0**	46	56	43	——	2	——	6	1	9
**3.0 - 3.5**	8	25	8	——	——	——	——	——	——
**3.5 - 4.0**	6	8	——	——	——	——	——	——	——
**4.0 - 4.5**	1	2	——	——	——	——	——	——	——
**> 4.5**	——	——	——	——	——	8	——	——	——
**TOTAL**	250	282	176	10	18	19	61	1	39

Table S2. *PDB ID codes for proteins containing PTM-modified residues with a 3.5 Å resolution cutoff.*

PTM	PDB ID
SEP	1ATP;1L3R;1APM;1FMO;1CMK;1BKX;1BX6;1JBP;1J3H;1LWO;1KHX;1OVA;1KKM;1I7W;1JLU;1SVG;1RDQ;1Q8U;1Q8W;1STC;1SMH;1REK;1SZM;1SVH;1SYK;1U7F;1YDR;1U7V;1U5R;1XJD;1UHG;1UJK;1VEB;2BVA;1ZEF;1ZEB;1Z8D;2BIK;1YHS;1YDS;1YDT;2C1A;2C1J;2CPK;2C1N;2C1B;2ERZ;2CDZ;2GNG;2GNL;2FEP;2GNF;2GLQ;2GNJ;2GFC;2GPA;2GNH;2GNI;2JDV;2OEN;2OBJ;2NZV;2JED;2JFL;2NRU;2JDT;2NZU;2J0I;2QCS;2OIB;2QLL;2QVS;2OIC;2QUR;2Q0N;2OID;2UVY;2UVX;3AGM;2XIX;2VNY;2XIY;2UVZ;3AMA;2VNW;2XCK;2VO0;2XIZ;3COJ;3AMB;3DNE;3BGZ;3BGM;3BWJ;3BGP;3DND;3DDW;3BGQ;3G2V;3FQR;3FHI;3F2A;3E8E;3EXH;3FQX;3FJQ;3FQU;3G2T;3HUF;3H9O;3IDC;3HRF;3HRC;3IQJ;3IDB;3IFQ;3IOP;3L9N;3NKX;3NAY;3KKV;3NUN;3IQU;3IQV;3L9M;3L9L;3L6F;3O8I;3OQM;3O7L;3OQN;3ORZ;3NUS;3OTU;3ORX;3NUY;3NUU;3QAM;3OVV;3P0M;3OW3;3PVB;3QCS;3POO;3QAL;3Q4A;3OXT;3QD3;3SQD;3T7K;3T9I;3RCJ;3RWP;3QCX;3RWQ;3SZM;3TPE;3UIM;3TMP;3UAL;3UZD;3UEO;3ULZ;3TPV;3U3Z;3UBW;4BZN;3ZKF;3VQH;3X2W;3X2V;4C0O;4BZO;3V7D;4FIG;4FIE;4FIF;4FII;4FIJ;4DAU;4EUU;4FIH;4DG0;4DFY;4IAI;4IAK;4HPT;4IAZ;4IAY;4IAD;4HPU;4IAC;4IAF;4IGK;4JAX;4JDH;4JDJ;4KIK;4IHL;4JDK;4N6Z;4N6Y;4JDI;4O0V;4OTI;4OTG;4O46;4OTH;4O0Y;4PSI;4NTS;4O0X;4NU1;4QPM;4RRV;4RQV;4UJB;4RQK;4WB8;4Q9Z;4UJ1;4RMZ;4XS2;4YP8;4YO6;4ZTM;5ACK;5K7I;5K7G;4ZTL;4ZTN;5K75;5T1S;5T1T;5UPL;5UZK;5K72;5K76;5VED;5UPK;5TOS;5VEE;5VEF
TPO	1H1Q;1L3R;1JSU;1JLU;1JST;1FMO;1H1S;1H1R;1FOT;1JBP;1QMZ;1O6K;1Q8W;1O6L;1PKD;1Q8U;1RE8;1RDQ;1P5E;1SZM;1W98;1SVG;1SVH;1U9I;1SMH;1REJ;1STC;1REK;1SYK;2A1A;1ZRZ;2B2T;1YDS;1YDT;1YRP;1YDR;1XJD;2CPK;2ERZ;2CCI;2ERK;2CCH;2C1B;2C6T;2C1A;2GBL;2G9X;2GNG;2GNF;2GFC;2JDV;2JFL;2GNI;2GNJ;2JDT;2IW6;2JDS;2GNL;2JDR;2GNH;2QCS;2JGZ;2NRU;2OIC;2NRY;2JFM;2OIB;2Q8Y;2OID;2O8Y;2QVS;2UZD;2VNW;2QUR;2UVX;2VNY;2VAG;2UVZ;2UVY;3A8X;2WMB;3A7J;3AGM;3A7H;3A8W;3AL3;2WMA;3A7G;3A7I;3AMB;3BLR;3BHT;3BLH;3BHV;3BHU;3AMA;3BLQ;3COM;3CKX;3D0E;3BZI;3CQW;3C4W;3BWJ;3CQU;3FHI;3DNE;3DVL;3DDP;3DND;3DOG;3E87;3E5A;3E8E;3DDQ;3FXZ;3K0E;3K0C;3K0A;3IDC;3FJQ;3IDB;3MI9;3K2L;3OJY;3O7L;3K09;3L9N;3L9M;3L9L;3MVJ;3POO;3PVB;3OW3;3P0M;3QAL;3OOG;3OXT;3QHW;3QAM;3OVV;3QKK;3UIM;3TN8;3TNH;3TXO;3TNI;3UIG;3TNW;3UEO;4BCF;4A0N;4BCG;4BCH;3UOT;3ULZ;4A0J;3UNN;3X2W;3X2V;4BCI;4C4F;4BCN;4BCK;4BCQ;4BCM;4BCO;4C4G;4BCJ;4BCP;4CRS;4CFU;4CXA;4CFW;4CEG;4CFV;4CFE;4CFH;4CFM;4CFN;4EOJ;4DUG;4DC2;4EOK;4DAW;4ELJ;4EOI;4EKL;4EOL;4EOR;4EOP;4EOO;4EON;4EOS;4EQC;4EOQ;4EWQ;4EOM;4IAZ;4IAC;4GLR;4IAK;4I3Z;4IAF;4IAD;4HPU;4IAI;4IAY;4OR5;4OTG;4IMY;4IZA;4NTS;4IJM;4NST;4OGR;4OTD;4QBS;4QMM;4QML;4QFG;4QMN;4QMO;4QMS;4QMP;4QFR;4QMQ;4QMU;4QMW;4QMX;4QMT;4RMZ;4RA4;4QMV;4QNA;4QMY;4QMZ;4ZTN;4XS2;4ZTM;4YO6;4YP8;4WB8;4UJB;4ZHX;4ZTL;4U8Z;5K72;5K7G;5K76;5K7I;5K75;5T1T;5T1S;5L2W;5KQ5;5UZK;6C9G;6ATH;5T5T;5V60;6BDL;5V61;6BG2;1APM;8H6P;1CMK;1ATP;1BX6;1BKX;8H6T;6C9J
PTR	3CLY;3BUX;3C7Q;3EB0;3BYO;3DQW;3BUW;3BYM;3CD3;3KCK;3EYH;3F7Z;3FUP;3F5P;3KVW;3KMM;3KRR;3LCK;3LXN;3KXZ;3MXY;3LPB;3MAZ;3LXP;3RVG;3SRV;3SAY;3PFV;3R7O;3NNX;3TJC;3OP0;3TJD;3VRO;3ZEW;3VRZ;3VRY;3VRP;3U3Z;3VRR;3ZEP;4CKJ;3ZUV;4AFJ;3ZMM;4CKI;4A4C;3ZNI;4AZF;3ZRK;4AQC;4E4L;4E5W;4E4M;4DIT;4EHZ;4E4N;4E6Q;4D1S;4E6D;4EI4;4F09;4GFM;4FK6;4GVC;4GFU;4HGE;4GPL;4I5C;4F08;4IVA;4IFC;4IZA;4IIR;4IAN;4IJP;4IVC;4IVD;4IWD;4IVB;4J1R;4K11;4NCT;4JMG;4LUE;4JI9;4JIA;4K6Z;4JMH;5A3X;4R3P;4R3R;4RXZ;4S0G;4Z16;4V0G;4QUM;5TQ6;5A54;5A4T;5A4L;5AMN;5CZI;5TQ3;5A4Q;5AIK;5AEP;5V60;5TQ7;6C7Y;5V61;5WEV;6CZ2;5USY;1FMK;1F1W;1AYA;1FBV;1AYB;1AYC;1I3Z;1PKG;1NZV;1GAG;1NZL;1LCJ;1O9U;1KSW;1LCK;1SPS;1QPJ;1SHA;1RQQ;1QPE;1QCF;1TRN;1UUR;1ZFP;1UUS;1YVJ;1YVH;2B7A;1YWN;2AUH;2B4S;2C0O;2GQG;2C0I;2H8H;2C0T;2ERK;2CBL;2HDX;2HCK;2IVV;2IVT;2HMH;2IUH;2IVU;2OFU;2OH4;2J0L;2VIF;2PTK;2Q8Y;2SRC;2QOQ;2X2L;2QON;2PVF;3BUN;2ZYB;3BUO;3ANR;2X2M;2Z8C
M3L	1CSX;1CIG;1CRH;1CRI;1CSW;1CSU;1CRG;1CIH;1CSV;1CRJ;1IRV;1PDQ;1KNE;1IRW;1CTY;1KYO;1CTZ;1PRW;2B2W;1RAP;2B2T;2B2U;1RAQ;1YCC;2X4X;3AVR;2X4W;2YCC;2X4Y;3G7L;3LQJ;3K

	QI;3CX5;3N9P;3U5O;3N9Q;3U5N;3N9N;3N9O;3QBY;4EZH;4IUR;3U5P;4N4I;4N4H;4MZH;4NW2;4LXL;4MZF;4MZG;6C4W;4V2V;6AT0;6ASZ;4V2W;1CHI;1CHH;1CIE;1CCR;1CHJ;1CIF
ALY	4LLB;4DNC;3U5P;3UVY;3UVX;4N4F;4QUT;3UVW;4N3W;4QUU;1SZC;4QYL;4QYD;2B5G;2H4F;1SZD;2GIV;2H2D;2E3K;2C1J;2QQF;2QQG;2OU2;2OD9;2OD7;2H4H;2I2Z;2YBG;2Y0M;3JVK;3GLR;3QZT;3JR3;3QZS;3U5N;3TOA;3U5O;3TOB;3QZV

Table S3. *Side-chain angle definitions for PTM-modified residues.*

	χ_1	χ_2	χ_3	χ_4	χ_5
**SEP**	N-CA-CB-OG	CA-CB-OG-P	CB-OG-P-O1P	————	————
**TPO**	N-CA-CB-OG1	CA-CB-OG1-P	CB-OG1-P-O1P	————	————
**PTR**	N-CA-CB-CG	CA-CB-CG-CD1	CE1-CZ-OH-P	CZ-OH-P-O1P	————
**M3L**	N-CA-CB-CG	CA-CB-CG-CD	CB-CG-CD-CE	CG-CD-CE-NZ	CD-CE-NZ-CM1
**ALY**	N-CA-CB-CG	CA-CB-CG-CD	CB-CG-CD-CE	CG-CD-CE-NZ	CD-CE-NZ-CH

Table S4. *Number of successful conformer builds with different rotamer libraries for each case of a protein containing intrinsically disordered protein regions.*

	**H4K9me3**	**UFD1**
**Unmodified**	1348	2009
**Bb. dp.**	854	1963
**Bb. indp.**	1266	2227
**SIDEpro**	1192	1998
**Rosetta (clash filtered)**	1359	1256

Table S5. *RMSDs of repacked PTM-modified residues using different rotamer libraries to experimental structures.* The numbers of PDB files evaluated are shown in parenthesis. SIDEpro does not support ALY packing.

**PTM Library**	**ALY** (31)	**M3L** (57)	**SEP** (226)	**TPO** (256)	**PTR** (156)
**BD-rotamers**	0.49 ± 0.28	0.96 ± 0.67	0.67 ± 0.53	0.76 ± 0.40	1.06 ± 1.29
**BI-rotamers**	0.96 ± 0.99	1.01 ± 0.50	0.92 ± 0.69	1.13 ± 0.88	1.56 ± 1.94
**SIDEpro**		1.20 ± 0.73	1.24 ± 0.77	1.06 ± 0.90	1.78 ± 1.68
**Rosetta**	1.40 ± 1.19	1.77 ± 0.67	1.66 ± 0.35	1.36 ± 0.47	1.86 ± 1.14

Supplementary Figures

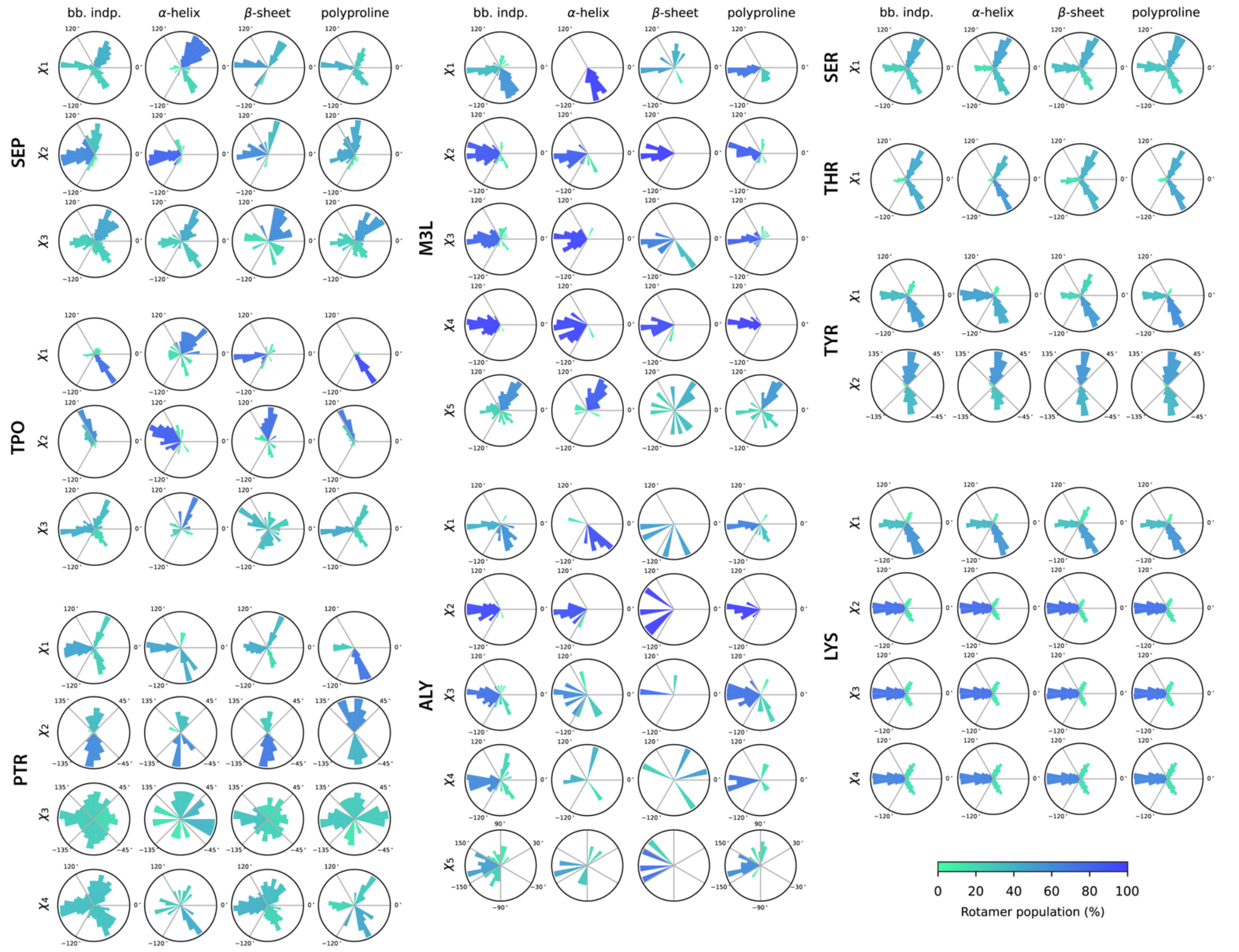

Figure S1. *Polar torsion angle histograms and rotameric bin populations for PTM-modified residues in major ϕ/ψ regions for folded proteins.* ϕ/ψ regions are defined as α-helix: $-105° \geq \phi > -45°$, $-60° \geq \psi > 30°$; β-sheet: $\phi < -105°$, $\psi \geq 90°$; polyproline: $-105° \geq \phi > -45°$, $\psi \geq 105°$. The backbone independent (bb. indp.) columns are based on all ϕ/ψ regions. Histograms are normalized such that the total area sums to 1. The distributions for canonical amino acids are calculated from SidechainNet's CASP12 dataset with 30% thining[1].

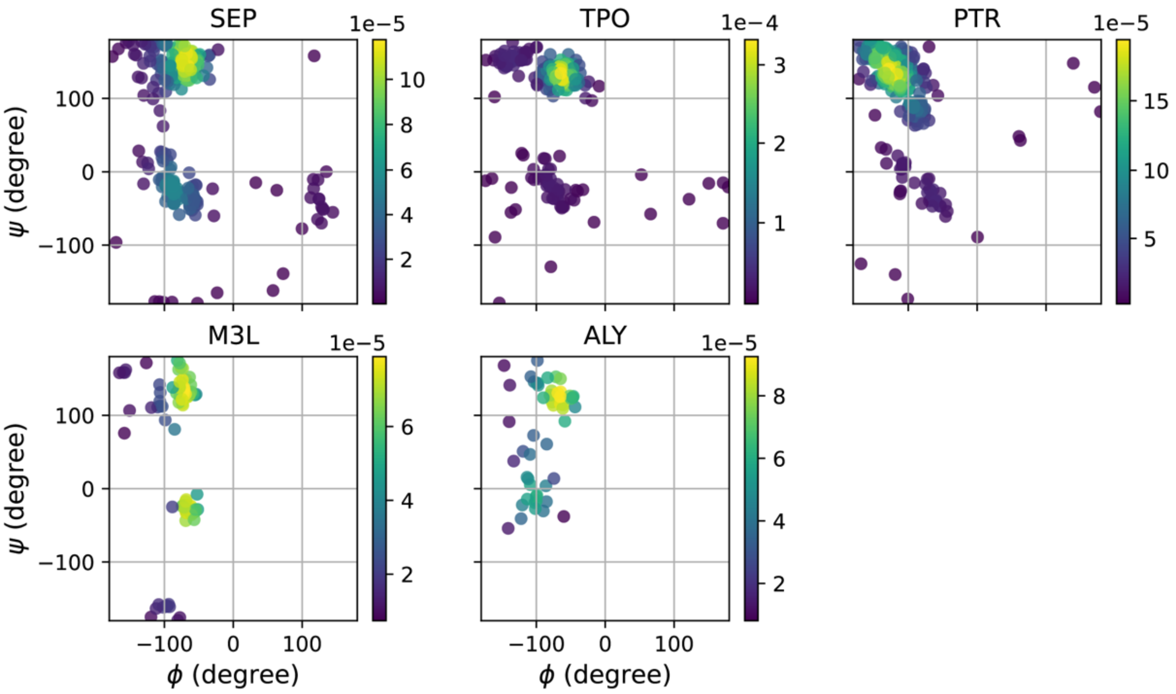

Figure S2. *Ramachandran plots for the curated PTM-modified residues.*

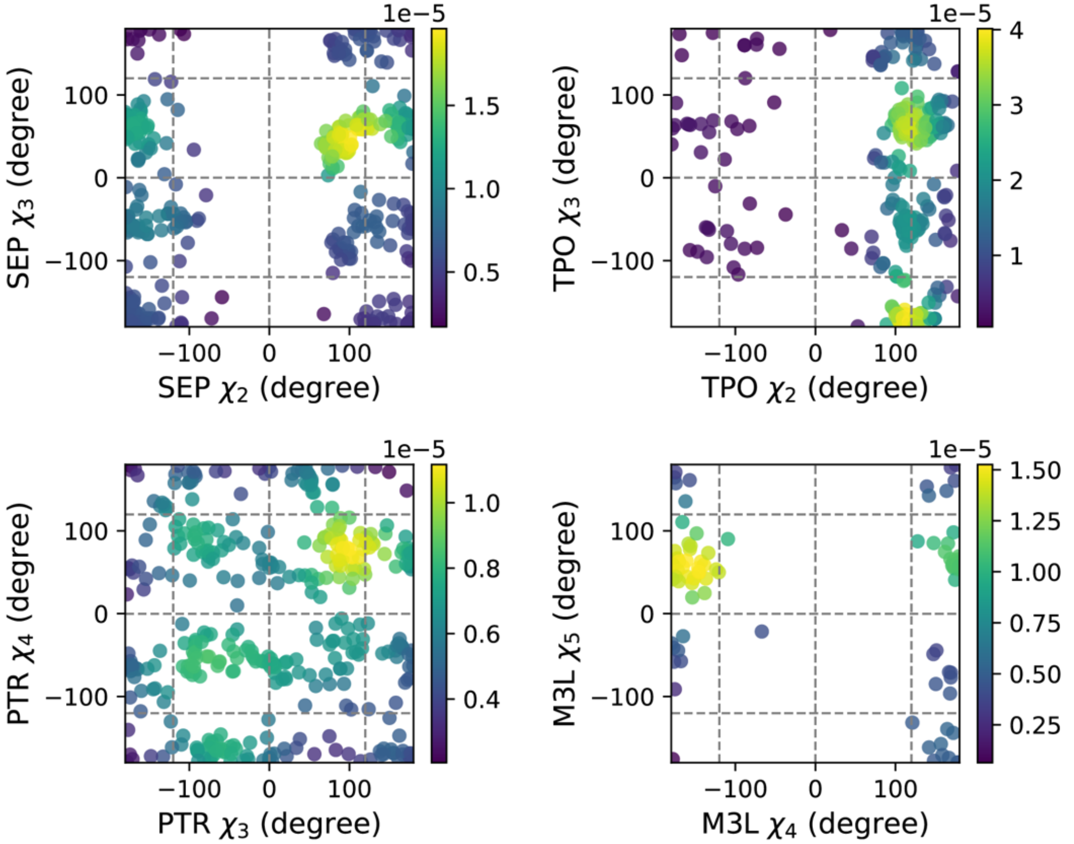

Figure S3. *Terminal torsion angle distributions for the curated PTM-modified residues.* Rotameric bin definitions for tetrameric (sp^3 bond hybridization) conformations are marked in gray dash line.

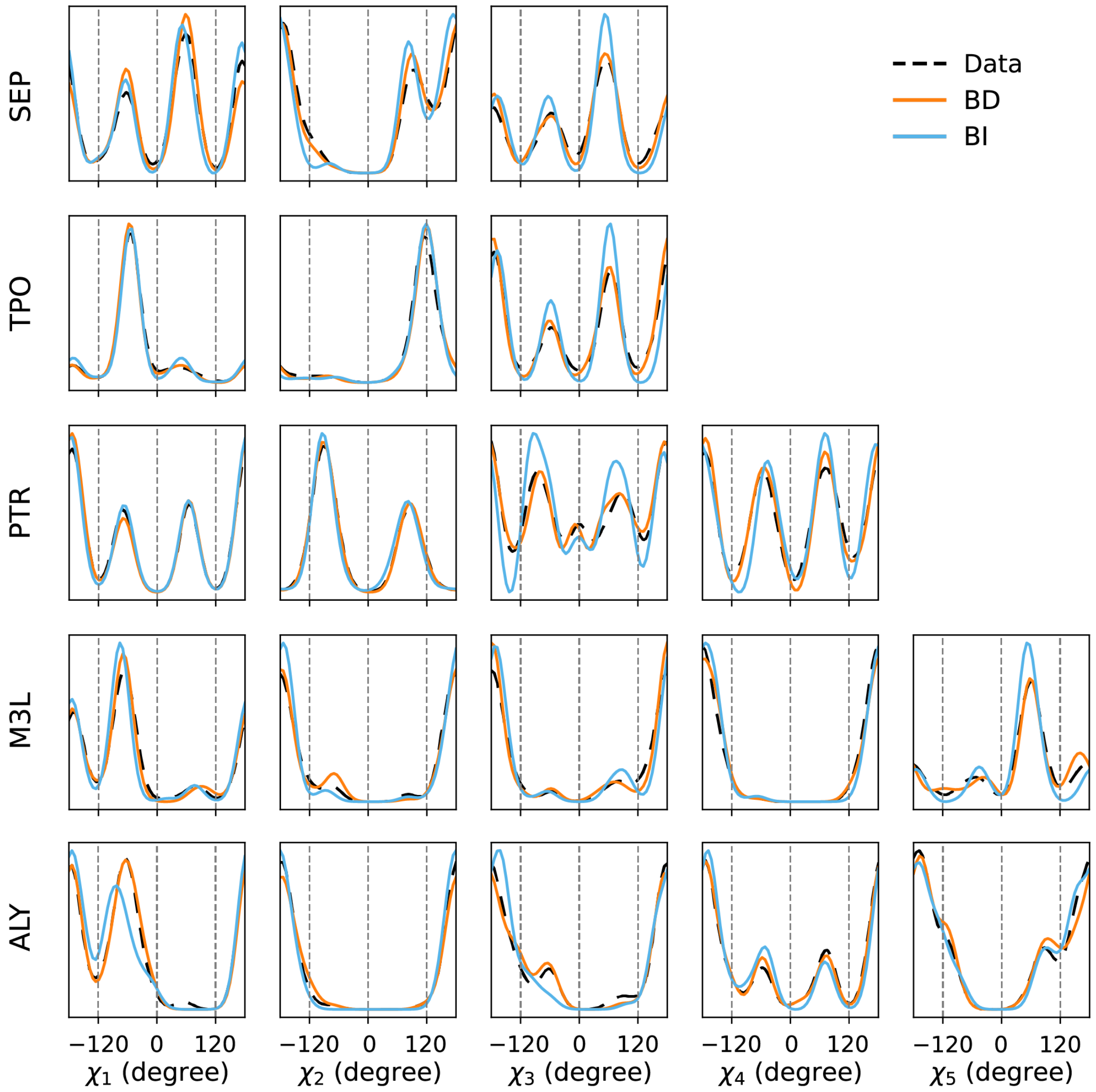

Figure S4. *Comparison of rotamer probability distributions for PTM-modified residues from curated PDB data and constructed libraries.* All angles are sampled using the same set of ϕ/ψ, and probability densities are estimated with von Mises kernel.

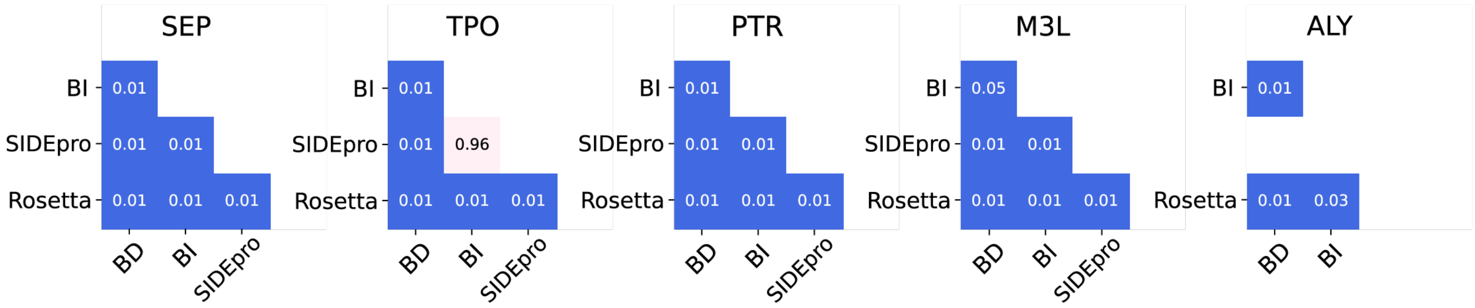

Figure S5. *Wilcoxon signed-rank tests for the RMSD distributions of repacked PTM-modified residues to experimental structures.* The one-tailed p-values smaller than 0.05 indicate that the of median of the pairwise difference between the bottom and left distribution is smaller than 0 with a 95% confidence level (highlighted in blue).

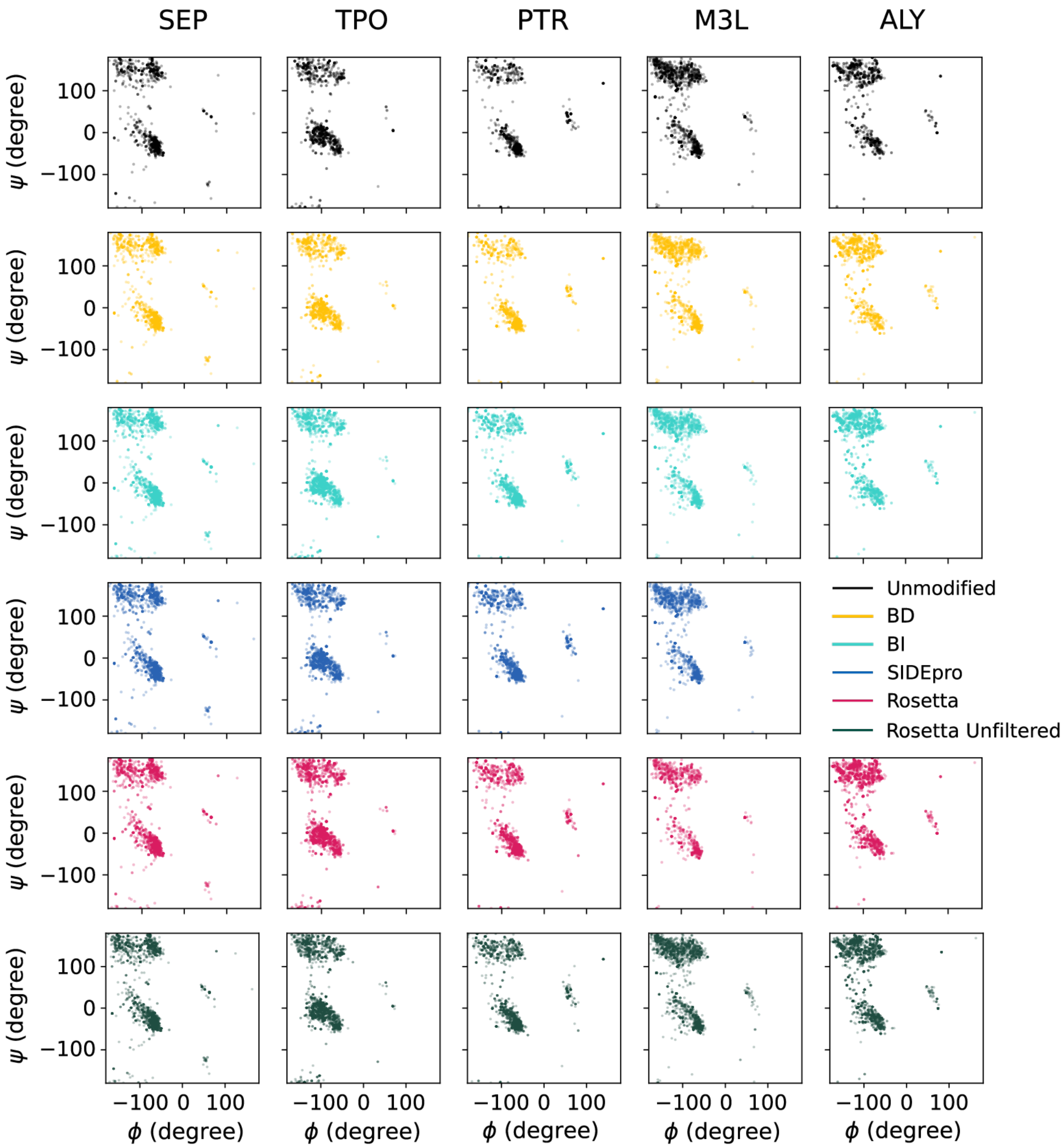

Figure S6. *Ramachandran plots of PTM-modified residues in the generated IDR-containing Histone H3 and UDF1 conformers with IDRs using different rotamer libraries.*

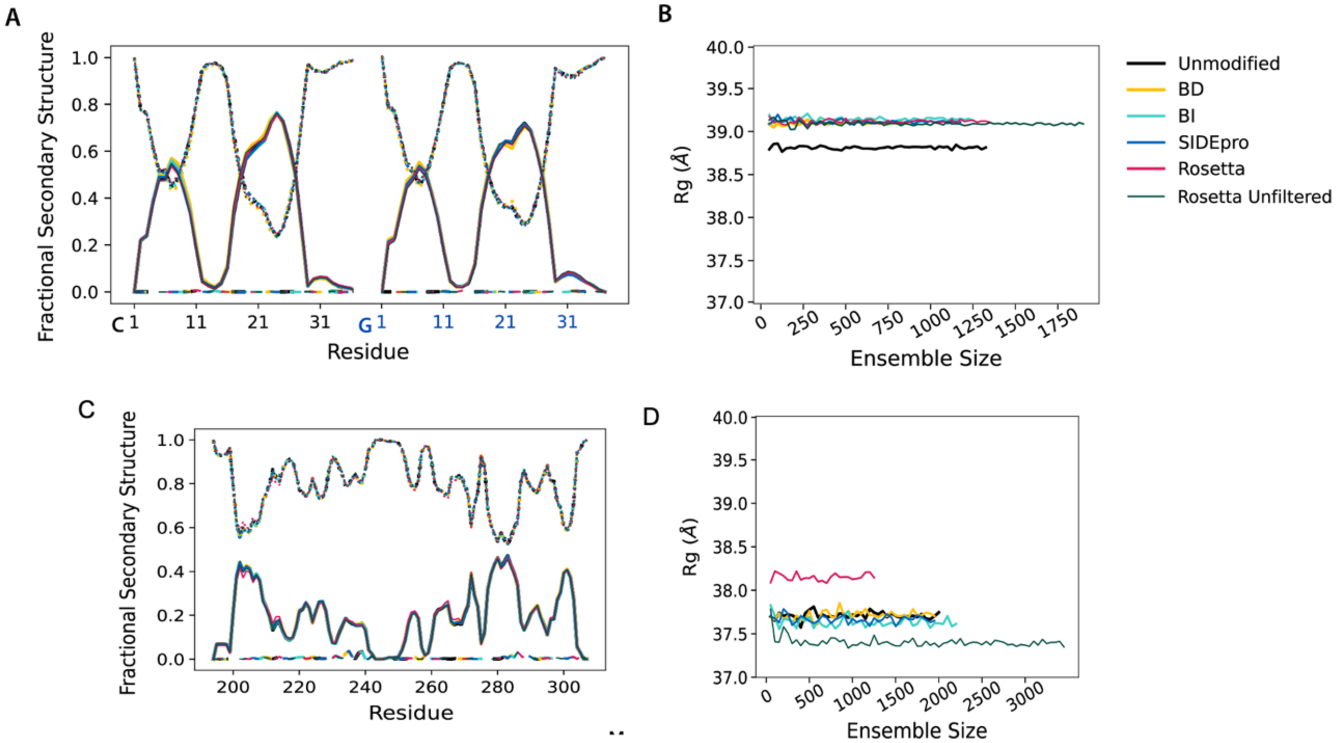

Figure S7. *Histone H3 and UDFI conformers generated with different PTMs libraries.* Fractional secondary structure propensities for (A) histone H3 N-terminal IDRs on chain C and G and (C) UDFI. Secondary structures were calculated using DSSP. Helices are shown in solid line, loop in dotted line and β-sheet in loosely dashed line. Mean radius of gyration over ensemble size, calculated from an average of 30 randomly selected ensemble for (B) histone H3 N-terminal IDRs on chain C and G and (D) UDFI

Reference

1 King, J. E. & Koes D. V. Sidechainnet: An all-atom protein structure dataset for machine learning. *Proteins: Structure, Function, and Bioinformatics* **89**, 1489–1496 (2021).
